# The Campaign against Syphilis: An Anticipation of the Royal Commission's Report

**Published:** 1914-02-28

**Authors:** 


### THE CAMPAIGN AGAINST SYPHILIS.

### An Anticipation of the Royal Commission's Report.

As the result of the increasing interest manifested by the public and the profession in the growing danger of luetic infection to the community at large, a Royal Commission has been appointed. Most Commissions of this sort mean the total shelving of the questions with which they have been commissioned to deal. This one, too, may possibly leave the problem very much where it found it: if it ventures to interpret its terms of reference strictly it will doubtless think its duty done when it has reported on the incidence of syphilis in this country, on the available means of treatment, on new methods, and on matters of subsidiary importance. But we imagine that the members of the Commission take a wider view of their responsibilities, and that they will act up to their view. That being the case, it is to be anticipated, with some degree of confidence, that the Commission will finally report on certain means which may be taken to stem the spread of the disease, and to safeguard the community as a whole.

The theoretic arguments in favour of limiting the danger by notification, inspection, and compulsory examination of suspects are well known to the public and to the Government. But what neither the Government nor the public know much about is the quiet, useful work which is being done by voluntary effort. Mr. Lane gave some indication to the Commissioners of the lines upon which this voluntary work is being organised by the London Lock Hospital at its night clinic. This description of methods of attack and their results will not be without influence upon the Commission, and if we may be permitted to prophesy, we should say that one of the recommendations of the Commission will be that similar work should be encouraged elsewhere, either by voluntary and co-operative effort, or, if nothing else can be done, then by the municipalities and by the Government.

Along these lines municipal effort has already crystallised in other countries, notably in South America. It is said, however, that such municipal effort has been a failure, largely owing to the want of public interest in the campaign. Perhaps that will be the case here, but there are indications that we are now very much in earnest. The National Insurance Act, for instance, has brought into the light of publicity just those points in regard to the syphilitic which were elicited when the German Acts came into operation, and the large sick clubs found that they were face to face with heavy expenses entailed by the incidence of syphilis in their members. Our own societies will probably profit by the lessons learnt in Germany, and try to co-operate with whatever efforts are made to deal with the problem along the lines we have indicated. The question of finance is relatively a small one; at present syphilis, as an "economic disease," is probably responsible for the loss to the working man of more wages and time than is tuberculosis. It is responsible to the community, directly and indirectly, for loss along other lines. The increase in insanity, in nervous disorders, in feeble-mindedness, cannot wholly be ascribed to the influence of syphilis, but there can be little question that the disease has something to do with this increase.

Already abroad the first attempt to deal with the question on lines of communal utility has been instituted. Hamburg and Bremen have decided to establish a special clinic, to be known as "Fuersorgestelle fuer Syphiliskranke," in which members of the various sick clubs, corresponding to our own National Insurance societies, shall have the benefit of expert diagnosis and advice. Treatment is not contemplated as yet, but will doubtless follow as a matter of course later on. All that is attempted is to establish a centre at which the man who supposes himself to be infected shall be able to get support of his private diagnosis or reassurance that he is free from infection. Those who are infected will received printed information regarding the general dangers they have to guard against, and special directions applicable to each individual case. The institution has been decided upon at a general conference attended by the representatives of the large sick clubs, the medical staffs of the large municipal hospitals in the Hanse cities, and delegates from the health committees of the municipalities. The municipality is to pay for the clinic, but the clubs will make grants in aid. It was opened on January 1 of the present year, and it will, no doubt, be of great utility to the community and lead, in course of time, to the establishment of similar models elsewhere.

Some such centre, or many of them, we should like to see established in this country. It is obvious that something will have to be done to deal with the growingly large percentage of infected persons in our midst. Already we possess, in the hospitals, the expert help which will be needed to make such a clinic, or series of clinics, a success. It is for the hospitals to recognise their responsibilities in the matter and attempt to start such clinics now when public interest in the matter is acute.

Already we are informed that two London hospitals have the matter under consideration. The London Hospital is starting such a clinic, and Guy's Hospital is about to follow suit. These are encouraging signs of the interest that the hospitals are taking in the matter; it is an added proof, if proof were wanted, of the fact that our voluntary hospitals are worthy of the trust which the public places in them. By shouldering vigorously their responsibilities in the matter and dealing with the question now, it is obvious that they will do an immense service to the public at large. One or two points, however, they would do well to bear in mind. One is that the clinics must be properly and expertly staffed. There must be no attempt to stint them, to place them in charge of young doctors who are not yet expert at diagnosis, and who, with the best will and intentions in the world, are incapable of dealing in the best possible manner with the class of patients who will frequent the clinics. Questions of differential diagnosis are often of great nicety; the experience of an old hand is required, and must be available. We have nothing to say against the training of students in such clinics; such training must be given, and will be given, we have no doubt, but the clinics must be immediately under the control of experienced surgeons and physicians whose expert knowledge cannot be questioned.

Another point of primary importance is that these clinics should be night as well as day clinics. There are doubtless serious difficulties in the way of realising this ideal, but we think that they should be, and can be, overcome by proper administrative arrangements. The average working man cannot afford to give up his midday hour of rest nor any part of his working time to attend such a clinic; opportunity must therefore be afforded him to attend when he is able to do so, in the evening, on Saturday afternoons, and, perhaps, early in the morning before he goes to work. Such an evening clinic is already in being in London, and has been found to be highly useful. We should like to see the example of the London Lock Hospital followed by the large general hospitals. While we are awaiting the report of the Royal Commission we may at least take time by the forelock and start such clinics. Their usefulness to the community it would be difficult to overestimate, while their helpfulness to the individual sufferers is generally and widely acknowledged.

